# Sensorimotor faculties bias choice behavior

**DOI:** 10.3389/fpsyg.2025.1432996

**Published:** 2025-03-28

**Authors:** Jan Kubanek, Lawrence H. Snyder, Richard A. Abrams

**Affiliations:** ^1^Department of Biomedical Engineering, University of Utah, Salt Lake City, UT, United States; ^2^Department of Neuroscience, Washington University School of Medicine, St. Louis, MO, United States; ^3^Department of Psychology and Brain Sciences, Washington University in St. Louis, St. Louis, MO, United States

**Keywords:** perceptual decision-making, free choice, embodied cognition, hand dominance, auditory system, right ear advantage

## Abstract

Decision-making is a deliberate process that seemingly evolves under our own volition. Yet, research on embodied cognition has demonstrated that higher-order cognitive processes may be influenced, in unexpected ways, by properties of motor and sensory systems. Here we tested whether and how decisions are influenced by handedness and by asymmetries in the auditory system. Right- and left- handed participants performed an auditory decision task. In the task, subjects decided whether they heard more click sounds in the right ear or in the left ear, and pressed a key with either their right or left index finger, according to an instructed stimulus-key assignment (congruent or reversed). On some trials, there was no stimulus and subjects could choose either of the responses freely. When subjects chose freely, their choices were substantially governed by their handedness: Left-handed subjects were significantly biased to make the leftward choice, whereas right-handed subjects showed a substantial rightward bias. When the choice was governed by the sensory stimulus, subjects showed a rightward choice bias under the congruent key assignment, but this effect reversed to a leftward choice bias under the reversed key assignment. This result indicates a bias toward deciding that there were more clicks presented to the right ear. Together, our findings demonstrate that human choices can be influenced by properties of motor and sensory systems.

## 1 Introduction

Choice behavior is a hallmark of higher-order cognition. During decision-making, subjects weigh the evidence that supports each alternative, and choose the alternative that is associated with a better outcome (Gold and Shadlen, 2007). This process is generally assumed to function deliberately, independently of particular properties of the systems that provide the input to or the output from this process.

Traditional models of cognitive function posit that processes involving higher-order cognition are separable from material and motor processes of the body (Markman and Dietrich, 2000; Wilson, 2002; Clark, 1999). According to these models, sensory modalities supply information to a centralized cognitive processing module. This module computes an outcome, such as a decision, and passes that outcome along to the motor system to trigger a desired action. The information-processing approach to model cognitive processes has over the past several decades dominated cognitive science (Markman and Dietrich, 2000; Wilson, 2002; Clark, 1999), artificial intelligence (Barsalou, 2008; Ghazanfar and Turesson, 2008), behavioral economics (Tversky and Kahneman, 1981), and systems neuroscience (Schall, 2002; Gottlieb, 2007).

This dominant information processing and sequential model, however, has recently been criticized. The critique targets the fact that the model reduces cognitive processing to the flow of information between dedicated processing modules, thus leaving little room for physical and motor aspects of the body to participate in cognitive processing. Recent work on embodied cognition by scholars across disciplines including philosophy, psychology, cognitive science, and artificial intelligence directly challenges this sequential model, suggesting instead that material properties of the body and aspects of motor behavior may be engaged in an ongoing fashion to support and shape higher-order cognitive processing (e.g., Barsalou, 2008; Clark, 1998, 1999; Pfeifer and Bongard, 2006; Thelen et al., 2001). In particular, in numerous behavioral experiments, embodied cognitive sciences have demonstrated that many seemingly abstract cognitive processes—such as, interpreting emotion (Niedenthal, 2007), processing language (Fischer and Zwaan, 2008) and numbers (Domahs et al., 2010), memory retrieval (Dijkstra et al., 2007), or decision-making (Gold and Shadlen, 2000, 2007; Selen et al., 2012; Kubanek et al., 2013)—are tightly associated with sensorimotor elements of the body. With respect to decision-making, neuroscientists have recently suggested that variables related to perceptual decisions, both internal and external, are detectable in motor circuits (Gold and Shadlen, 2000, 2007; Selen et al., 2012; Kubanek et al., 2013), and influence behavioral output (Treviño, 2014; Treviño et al., 2020; Treviño and y León, 2020; Treviño et al., 2021). This opens the possibility that perceptual decisions may be influenced by particular properties and asymmetries of motor (Hicks and Kinsbourne, 1976; Corballis, 1997; Bryden et al., 1994; Bishop et al., 1996; Gabbard et al., 1998; Calvert, 1998; Stins et al., 2001) and sensory (Kimura, 1961a,b; Broadbent and Gregory, 1964; Knox and Kimura, 1970; Kimura, 2011) systems.

It has been hypothesized that the degree of embodiment depends on the degree of dissociation of the peripheral from the cognitive systems (Barsalou, 2008; Clark, 1998, 1999; Pfeifer and Bongard, 2006; Thelen et al., 2001). In many of the previous studies, this dissociation did not receive sufficient attention, and thus embodiment could be expected as a mere consequence of task design.

Aiming to maximize the dissociation between decision and peripheral systems, we engaged subjects in a decision task that is based on defined quanta of sensory evidence while resulting in simple motor responses (Xie et al., 2024; Kubanek et al., 2015, 2013). In this task, subjects perceive discrete quanta of auditory evidence for a left and a right choice, which engages the sensory, auditory system. Subjects accumulate these quanta and make a comparison, which engages higher-order cognition. Finally, subjects communicate their response with a left or a right index finger, which engages low-level motor representations. The sensory and motor aspects involved in this task are defined, simple, and stereotypic, and thus are not expected to influence the decision process itself. Surprisingly, we found that decision-making in this task was biased by sensory and motor aspects of the body.

## 2 Methods

### 2.1 Subjects

Fifty-four Washington University undergraduate students (37 females, 17 males), aged 18 to 21 (mean 19.2) participated in this study. All subjects gave an informed consent. The subjects were healthy and had normal hearing capacity, which is evidenced by a standard psychometric curve observed in this task, as in Kubanek et al. (2013); Xie et al. (2024). Subjects participated for class credit.

### 2.2 Apparatus and procedure

Subjects sat in a comfortable chair 70 cm in front of a flat-screen monitor. Subjects wore headphones (MDR-V600, Sony), which presented a stereo auditory stimulus (see Auditory stimulus). The volume in the left channel was set to the same level as the volume in the right channel. The subjects' hands were comfortably positioned at a computer keyboard with the left index finger placed over the left Alt key and with their right index finger placed over the right Alt key. The control of the experimental design was accomplished using a custom program written in Matlab (The Mathworks, Inc., Natick, MA).

**Figure 1:**
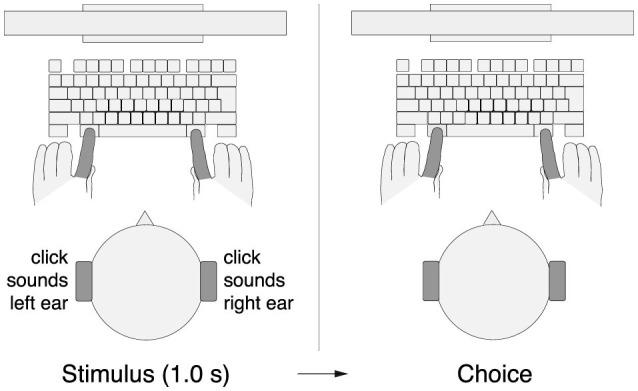
Perceptual decision task. Subjects listened to a binaurally presented auditory stimulus that comprised a 1.0 s train of Poisson-distributed click sounds (Methods). Following the stimulus presentation, subjects pressed either the left Alt key with their left index finger or the right Alt key with their right index finger, depending on a particular key assignment.

Each trial started with the presentation of a red fixation cross, 2 degrees in size. Subjects were instructed to fixate at the center of the cross. At the same time, subjects were presented with a stereo auditory stimulus (click sounds, see Auditory stimulus), 1.0 s in duration (Figure 1). After the stimulus had been presented, the fixation cross shrank to 1 degree and changed its color to green. This event cued the subjects to make a movement (choice). Subjects performed 2 blocks of 300 trials each, with a brief break in between. In the first block of 300 trials, subjects were instructed to press the left Alt key with their left index finger if they heard more clicks in the left ear and to press the right Alt key with their right index finger if they heard more clicks in the right ear. In the second block of 300 trials, this instructed key assignment was reversed. The first block was completed by all 54 subjects, the second block by all but one subject.

On 20% of the trials (randomly selected), no auditory stimulus was presented. When no sound was heard, subjects were instructed to choose either key (i.e., to either press the left key with the left index finger or the right key with the right index finger). The purpose of these trials was to study choice that is self-initiated by the subject.

If subjects responded prior to the green go cue or if they failed to indicate a response within 1200 ms after the go cue, the trial was considered invalid, and was aborted and excluded from the analyses. The type of error was indicated to the subjects in red, large-font text (“TOO EARLY,” “TOO LATE”). Overall, the proportion of valid responses was 95.6 ± 6.1% (mean ± s.d.) in the first block, and 96.4 ± 13.6% in the second block. A response was immediately followed by a display of a feedback. Specifically, a correct response was followed by the display of a green string that was randomly drawn from the set {+5*c*, +10*c*, +15*c*, +20*c*, +25*c*}. An incorrect response was followed by the display of a red string randomly drawn from the set {−5*c*, −10*c*, −15*c*, −20*c*, −25*c*}. The feedback was displayed for 0.5 s. The next trial started immediately following the offset of the feedback. The effect of the feedback was analyzed in a dedicated study (Kubanek et al., 2015).

### 2.3 Auditory stimulus

The auditory stimulus presented to each ear consisted of a train of brief (0.2 ms) click sounds drawn from a homogeneous Poisson process (Kubanek et al., 2013). Each train lasted 1.0 s. The stereo stimulus was composed such that the number of clicks presented to the left ear (*C*_*l*_) plus the number of clicks presented to the right ear (*C*_*r*_) summed to a fixed number *C*_*l*_ + *C*_*r*_ = Ω, Ω ∈ {25, 32, 39, 46}. The value of Ω was drawn randomly on each trial. We imposed this constraint to ensure that subjects had to attend to the click sounds in both ears. Stimulus presentation was also subject to the constraint that two consecutive clicks had to be separated by at least 5 ms. Furthermore, during pilot testing, subjects often claimed that they were biased toward the ear that presented either the first or the last click. To avoid such possible bias, the first and the last clicks in each stimulus occurred in both ears simultaneously, at time 0.0 s and 1.0 s, respectively. Thus, each ear received at least 2 clicks, and at most Ω−2 clicks. We generated ten random versions of each of the 130 possible combinations of *C*_*l*_ and *C*_*r*_, and loaded the corresponding files into the memory of the custom program prior to the start of each session. The trials were presented in random order. The logistic fit to the behavioral data were performed as in a previous study (Xie et al., 2024).

### 2.4 Online adaptive procedure

We set the difficulty of the task such that subjects make difficult, non-trivial decisions, with the goal to engage higher-order cognition. Specifically, we set the difficulty such that subjects were correct in approximately 60% of the trials (chance is 50% for this binary choice task). We achieved this using an adaptive staircase procedure (Kubanek et al., 2013). This procedure allowed subjects to perform close to the desired accuracy [first block: 61.9 ± 2.8% (mean ± s.d., *n* = 54); second block: 60.3 ± 8.7%].

### 2.5 Handedness score

When recruiting subjects, we encouraged the participation of left handed subjects, to obtain as balanced a proportion of right- and left- handed subjects as possible. After each subject completed testing, they answered a set of question that probed the subject's handedness. We used a set of questions based on the revised Edinburgh Handedness Inventory (Williams, 1986). This test returns a number between −100 (strongly left-handed) and +100 (strongly right-handed). The mean ± s.d. score over our subjects was +41.2 ± 57.0. We divided the subjects into three groups based on the handedness score. Subjects with a score higher than +33 were considered right-handed, subjects with a score less than −33 left-handed, and the subjects with a score between −33 and +33 were considered ambidextrous.

### 2.6 Statistical evaluation

Analyses were performed using a custom script developed in Matlab (The Mathworks, Inc.). All valid trials were included in the analysis, including during the flip of congruency. The dependence of choice proportion on handedness was assessed using linear regression. Contrasts between choice frequencies across subjects were assessed using two-tailed t-test; this was appropriate because the choice proportions were close to 50%, and the data were normally distributed. We validated that the underlying distributions were indeed normal using the Anderson-Darling test. Effects were considered significant in cases of *p* < 0.05. All test were two-tailed so that statistical analyses could detect an effect of either polarity.

## 3 Results

### 3.1 Choice behavior

**Figure 2:**
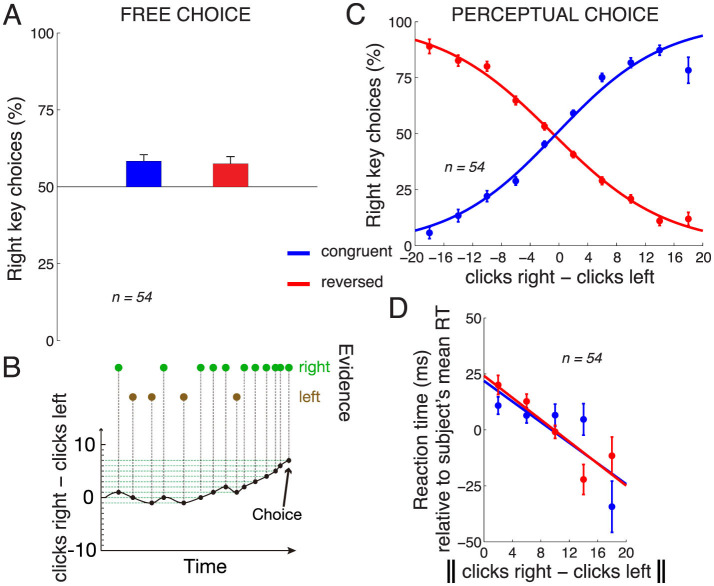
Choice behavior. **(A)** Mean ± s.e.m. proportion of rightward choices in the trials in which no stimulus was present and subjects chose freely. The data are shown separately for the congruent (blue) and reversed (red) response assignments. **(B)** Evidence accumulation during an example trial. Green/brown dots indicate auditory clicks presented to the right/left ears. **(C)** Mean ± s.e.m. proportion of rightward choices as a function of the difference in the number of clicks in the right and the left ear, separately for the congruent and reversed response assignments. The curves represent logistic fits to the 10 data points in each block. **(D)** Mean ± s.e.m. RT as a function of the absolute difference in the number of clicks in the right and the left ear, separately for the congruent and the reversed block. To control for differences in mean RT over the subjects (445 ± 123 ms, mean ± s.d.), the mean RT was subtracted from each RT value in each subject. The line is a fit to the 5 data points in each block. Congruent block, *n* = 54, reversed block, *n* = 53 subjects.

Figure 2A shows subjects' choice behavior when they could choose either response freely (during the 20% of control trials in which no stimulus was presented). As seen in the figure, on those trials, subjects showed a bias to choose the rightward key. In the congruent key assignment block, the mean proportion of rightward choices across the subjects was 58.3%, and this was statistically different from 50% [*t*_53_ = 3.85, *p* = 0.00032, two-tailed t-test; the data were normally distributed (Anderson-Darling test, *p* = 0.59)]. This bias was observed also in the reversed key assignment block [57.4%, *t*_52_ = 3.16 (one subject did not perform the reversed block), *p* = 0.0026; Anderson-Darling test *p* = 0.09].

When subjects made decisions based on the perceptual stimulus, their responses followed the given instruction (Figures 2B, C). Specifically, in the congruent block (blue), when subjects heard substantially more (e.g., 10 more) clicks in the right ear than in the left ear, subjects predominantly pressed the right key, and vice versa. When the instructed key assignment reversed, the choice behavior accordingly reversed (red). We quantified the choice behavior using logistic regression, as shown in the logistic fits in Figure 2C. We applied the logistic regression to the choice data of each individual subject. To determine whether the stimulus was a significant factor in guiding the subjects' responses, we measured the weight assigned to the click difference in this regression. This weight significantly differed from zero over the subjects (congruent block: mean weight 0.17 per click, *t*_53_ = 12.92, *p* < 0.0001, two-tailed t-test; reversed block: mean weight −0.15 per click, *t*_52_ = −15.27, *p* < 0.0001).

The amount of information in the stimulus may influence the time it takes subjects to produce a response, the reaction time (RT). We indeed found that the more information in the stimulus (greater difference in the number of the clicks between the two ears), the faster the subjects responded (Figure 2D). We quantified this relationship by fitting a line to this relationship in each subject, and measured the slope of the line. The mean slope over the subjects in the congruent block was −1.74 ms per click, and this slope significantly differed from zero (*t*_53_ = −2.61, *p* = 0.012, two-tailed *t*-test). In the reversed block, the mean slope was −2.85 ms per click (*t*_52_ = −3.99, *p* = 0.00021). Note that these numbers are averages of the slopes computed separately in each subject, compared to the slopes shown in Figure 2D, which represent, for visualization purposes, fits to data combined across all subjects.

### 3.2 Effect of handedness

**Figure 3:**
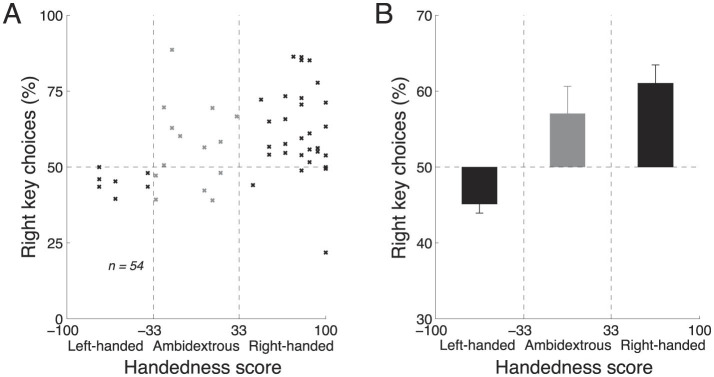
Handedness affects free choice. **(A)** Mean proportion of rightward choices as a function of each subject's handedness, in the trials in which there was no stimulus and so in which subjects chose freely. The vertical dashed lines segregate the effects in the left-handed, right-handed, and ambidextrous groups of subjects. **(B)** The mean ± s.e.m. effects computed using the data in **(A)**, separately for the left-handed, right-handed, and ambidextrous subjects. In **(A, B)**, data were pooled across the two blocks in each subject; *n* = 54 subjects.

We first investigated the effects of handedness and the key assignment during free choice, i.e., in the 20% of trials in which there was no stimulus and subjects could freely choose to press either the left or the right key. In these trials, there was no stimulus and we therefore averaged the choice proportions for each subject across the two blocks. We then plotted the average proportion of rightward choices as a function of handedness, separately for each subject (Figure 3A). The figure reveals that the proportion of rightward choices increases with the extent to which the subject is right-handed. When these data are fitted with a line, the slope of the line indicates a 15.0% change in the percentage of rightward choices over the range of handedness, and this slope significantly differs from zero (*t*_52_ = 2.35, t-statistic, *p* = 0.023, two-tailed). We further quantified the effects of handedness on choice for left-handed, ambidextrous, and right-handed subjects (Figure 3B). Left-handed subjects (handedness scores lower than −33) preferred to make leftward choices (mean proportion of rightward choices, 45.2%). In contrast, right-handed subjects (handedness scores higher than +33) preferred to make rightward choices (mean proportion of rightward choices, 61.1%). Ambidextrous subjects (handedness scores between −33 and +33) showed a tendency to choose the right key (mean proportion of rightward choices, 56.8%). Thus, when subjects make a choice based on their own deliberation, handedness is a significant factor in guiding the choice.

### 3.3 Effect of auditory processing asymmetry

Finally, we tested whether and how handedness and the key assignment affect choice during perceptual decisions, i.e., in the trials in which subjects' choices were guided by the auditory stimulus (Figure 2C top). In contrast to free choice, there was no significant modulation by handedness in either the congruent (slope: *t*_52_ = 0.49, *p* = 0.63, two-tailed *t*-test) or reversed (slope: *t*_51_ = −0.27, *p* = 0.79, two-tailed *t*-test) key assignment.

**Figure 4:**
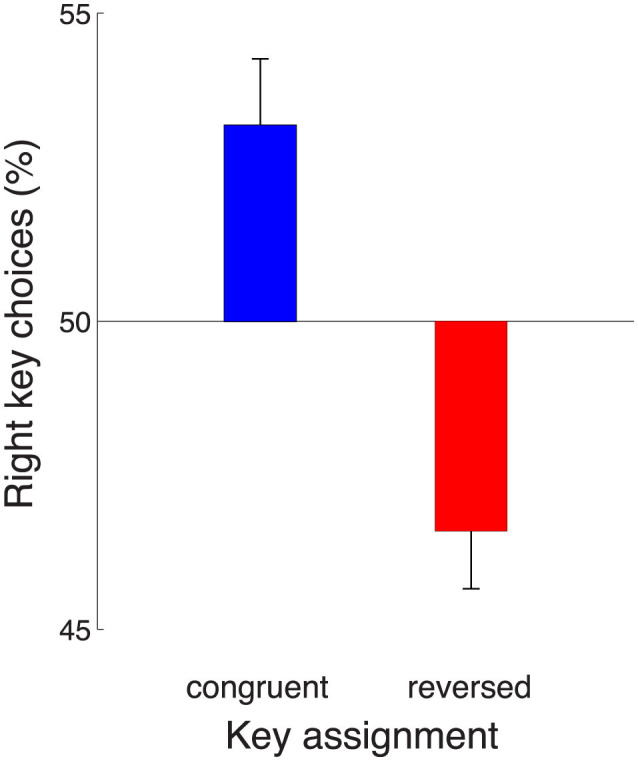
Auditory decision-making is affected by a right-ear enhancement. Mean ± s.e.m., over the individual subjects, proportion of rightward choices in the task in which subjects' choices were based on the auditory stimulus. The data are shown separately for the congruent (blue) and reversed (red) key assignments. The rightward choice preference (blue) reverses when the key assignment is reversed (red). This indicates an enhancement of the representation of sensory evidence presented to the the right ear. Congruent block, *n* = 54, reversed block, *n* = 53.

The effect of the key assignment on choice is shown in Figure 4. The figure reveals that in the congruent block of trials, subjects preferred to make, on average, a rightward choice (blue). This effect (mean 53.2%) significantly differs from 50% [*t*_53_ = 2.94, *p* = 0.0049, two-tailed *t*-test; the data were normally distributed (Anderson-Darling test, *p* = 0.16)]. This rightward choice preference could be either due to a movement-related effect or a sensory-related effect. In particular, in the congruent block, the effect may indicate an enhanced representation of the motor plan to press the right index finger, but it may also reflect an enhanced representation of the number of click sounds presented to the right ear. Reversing the key assignment provides a means to distinguish between these two possibilities. Intriguingly, when the key assignment reversed, the subjects' choice preference also reversed (red). Subjects now preferred to choose the leftward option (mean rightward choices, 46.6%) and this leftward choice bias was significant (*t*_52_ = −3.64, *p* = 0.00063; Anderson-Darling normality test, *p* = 0.36). The finding of a reversal of the rightward preference upon the reversal of the key assignment rules out a general rightward response bias. Instead, the effect indicates an enhanced representation of the click sounds presented to the right ear.

## 4 Discussion

This study demonstrates that free choice and perceptual decision-making are embodied even in a simple decision task. Specifically, we found that subjects' choices depend on motor- and sensory-related attributes of the decision-maker—the subject's handedness, and biases in the auditory perceptual system. Handedness substantially influenced subjects' choices on trials in which subjects freely chose whether to press a left key with a left finger or a right key with a right finger. On trials in which the choices were based on a stereo auditory stimulus, subjects' choices revealed a right ear-related enhancement and no handedness bias.

The study used simple motor responses and simple, defined auditory stimuli to dissociate peripheral aspects from higher-order cognition. If this dissociation was not performed, sensory or motor effects on cognition could be expected by default. For instance, if the input was based on face recognition, which is an inherent component of higher-order cognition, sensory and cognitive factors would likely be unsurprisingly intertwined. Similarly, if the output was a complex motor task that requires dexterity, an interplay with cognition would not be surprising. In the same vein, the decision task was chosen to provided a defined accumulation process—based on discrete quanta of evidence (Brunton et al., 2013), and to separate sensory input and motor output from the evidence accumulation process. Specifically, the task rested on a summation and a comparison of defined incoming quanta of sensory evidence. This summation and comparison are independent of specific motor outputs or any noise associated with the auditory inputs.

It has been shown that the selection of which hand to use to reach for an object is influenced by subjects' handedness (Bryden et al., 1994; Bishop et al., 1996; Gabbard et al., 1998; Calvert, 1998; Stins et al., 2001). Specifically, in these tasks and their variants, an experimenter systematically varies a particular property or location of an object within a subject's workspace. Subjects are then asked to reach for or manipulate the object using the hand of choice. It is commonly found that the relative frequency with which a hand is selected is a function of handedness: right-handed subjects on average prefer to reach for an object using the right hand, whereas left-handed subjects prefer to reach for an object with the left hand. In these studies, a reach for, a grasp of, or a manipulation of an object involves a relatively complex movement that requires dexterity and often further engages a higher-order computation that must weigh which hand is more suitable for or efficient in successfully completing the movement (Bryden et al., 1994; Bishop et al., 1996; Gabbard et al., 1998; Calvert, 1998; Stins et al., 2001). It is then perhaps unsurprising that to successfully perform such movements, subjects prefer to use the hand that they have been using predominantly for such purpose throughout their life (Serrien et al., 2006).

The motor responses constituted a defined key press using a finger. The left index finger was positioned over one key and the right index finger over another. In this state, subjects decided which of the keys to press. Left-handed subjects showed a bias in pressing the left key, whereas right-handed subjects showed the opposite bias (Figure 3). Given the relative simplicity of the movement, it is surprising to find that subjects' choices were affected by handedness. This suggests that the effect of handedness may extend beyond a choice of a dexterous movement; it may impact choices that involve a movement of the hand in general. This is supported by electrophysiological and interventional studies (Brasil-Neto et al., 1992; Kim et al., 1993; Stancák and Pfurtscheller, 1996; Schluter et al., 1998; Solodkin et al., 2001; Serrien et al., 2006) that report neural signatures of hand dominance in premotor and motor regions, regions that plan and execute both complex and simple movements.

Notably, the effect of handedness was observed in a task in which no stimulus was present. When the subjects' choices were guided by the perceptual stimulus, there was no significant effect of handedness on choice (and no handedness-based interaction). This is in line with findings made in dichotic word discrimination tasks in which handedness had no or minimal effect on subjects' judgements (Curry, 1974; Kimura, 1983). Thus, the influence of handedness on choice may be suppressed when a decision is guided by sensory information. Our data reveal that the prominent effect of handedness on choice is observed specifically during *self-initiated* choices.

When the subjects' choices were based on the stereo auditory stimulus, subjects showed a significant rightward choice bias with the congruent key assignment (Figure 4, blue). When the key assignment reversed, the effect reversed to a significant leftward bias (Figure 4, red). These two effects together indicate that there is an enhancement in the perception of the sensory information presented to the right ear. In this regard, there are findings of asymmetric representations of auditory information in the literature. In particular, in dichotic listening tasks, each ear is simultaneously presented with spoken words. In these tasks, subjects correctly identify more words presented to the right ear compared to words presented to the left ear (Kimura, 1961a,b; Broadbent and Gregory, 1964). However, interestingly, this effect is specific to verbal material; sounds that are not words, such as vocal and non-vocal environmental sounds, appear to show a weak reverse effect—a left ear superiority (Knox and Kimura, 1970; King and Kimura, 1972). The presence of a right ear advantage specifically in verbal tasks has led to the suggestion that the effect may reflect the lateralization of language processing to the left hemisphere, given the dominance of the crossed auditory pathways over the uncrossed pathways (Geschwind and Galaburda, 1984; Kimura, 2011). We found a right-ear advantage in a non-verbal task, a task that requires an accumulation of discrete quanta of auditory evidence over time (Brunton et al., 2013). Because the stimuli were non-verbal, the effect we report may have a different origin than the word-specific right ear advantage reported previously (Kimura, 2011).

An outstanding question is at which stage of the sensorimotor transformation handedness affected choice. Literature on the neural basis of handedness helps to provide an answer. Specifically, handedness generally appears to manifests in motor planning stages of the brain, including motor, premotor, and parietal cortices (Hammond, 2002; Gut et al., 2007; Schmitz et al., 2019; Brasil-Neto et al., 1992; Kim et al., 1993; Stancák and Pfurtscheller, 1996; Schluter et al., 1998; Solodkin et al., 2001; Serrien et al., 2006). Therefore, the handedness bias is likely superimposed on the choice process late during the decision formation. This bias could also emerge as late as during action planning. For instance, during difficult, ambiguous decisions, motor cortices representing both choices may be equally active, and handedness may provide the decisive advantage. This hypothesis could be tested using electrophysiological recordings. The motor (handedness) and sensory (right-ear advantage) effects in this study were independent since the response bias flipped polarity on reversing the contingency, and the mirroring was approximately symmetric with respect to 50%. Therefore, these two effects likely have independent neurophysiological sources.

The perceptual task used in this study may appear relatively simple, and it could therefore be argued that it may not involve higher-order cognition, which may influence the interpretation of the findings. Nonetheless, in this task, subjects are required to keep track of two auditory accumulators, compute a sum within each, and make an engaging comparison between the two. The difficulty was set to a high level (60% correct, relative to 50% correct due to chance). Moreover, a recently published study, which recorded neural signals directly from the cortex during this task (Xie et al., 2024), provides additional, neural evidence that the task engages higher-order cognition. The study found broad engagement of the cortex during this task, including parietal, premotor, frontal, as well as auditory and motor cortices. This broad cortical activation, including in frontal and parietal cortices, suggests that the task indeed engages cognitive processing. These aspects support the conclusion of the study that sensorimotor faculties have the capacity to influence high-order cognition.

The study has certain limitations. First, because right-handed subjects constitute a majority, there was an imbalance between right- and left-handed subjects. This limitation could influence the generalizability of the findings. And second, the study was not designed to systematically evaluate order effects, e.g., effects following certain patterns of choices.

In summary, we found that in a perceptual decision task, subjects' choices were influenced by their handedness and an enhancement of perceptual evidence presented to the right ear. When subjects were free to choose to make a simple finger movement with either hand, their choices were biased by handedness. When their decisions were based on a stereo auditory stimulus, the choices indicated a bias toward the right ear, and no effect of handedness. Thus, the seemingly deliberate process of making a simple choice can be partially embodied, skewed by asymmetries of the human motor and sensory systems.

## Data Availability

The raw data supporting the conclusions of this article will be made available by the authors, without undue reservation.

## References

[B1] Barsalou L. (2008). Grounded cognition. Annu. Rev. Psychol. 59, 617–645. 10.1146/annurev.psych.59.103006.093639 17705682

[B2] Bishop D. Ross V. Daniels M. Bright P. (1996). The measurement of hand preference: a validation study comparing three groups of right-handers. Br. J. Psychol. 87, 269–285. 10.1111/j.2044-8295.1996.tb02590.x 8673359

[B3] Brasil-Neto J. Pascual-Leone A. Valls-Solé J. Cohen L. Hallett M. (1992). Focal transcranial magnetic stimulation and response bias in a forced-choice task. J. Neurol. Neurosurg. Psychiat. 55, 964–966. 10.1136/jnnp.55.10.964 1431962 PMC1015201

[B4] Broadbent D. Gregory M. (1964). Accuracy of recognition for speech presented to the right and left ears. Quart. J. Exper. Psychol. 16, 359–360. 10.1080/1747021640841639226614048

[B5] Brunton B. W. Botvinick M. M. Brody C. D. (2013). Rats and humans can optimally accumulate evidence for decision-making. Science 340, 95–98. 10.1126/science.1233912 23559254

[B6] Bryden M. Singh M. Steenhuis R. Clarkson K. (1994). A behavioral measure of hand preference as opposed to hand skill. Neuropsychologia 32, 991–999. 10.1016/0028-3932(94)90048-5 7969872

[B7] Calvert G. (1998). Quantifying hand preference using a behavioural continuum. Laterality 3, 255–268. 10.1080/713754307 15513088

[B8] Clark A. (1998). Being There: Putting Brain, Body, and World Together Again. New York: MIT press.

[B9] Clark A. (1999). An embodied cognitive science? Trends Cogn. Sci. 3, 345–351. 10.1016/S1364-6613(99)01361-3 10461197

[B10] Corballis M. C. (1997). The genetics and evolution of handedness. Psychol. Rev. 104:714. 10.1037//0033-295X.104.4.714 9337630

[B11] Curry F. (1974). A comparison of left-handed and right-handed subjects on verbal and non-verbal dichotic listening tasks. Exper. Phonet. 3:410. 10.1016/S0010-9452(67)80022-4

[B12] Dijkstra K. Kaschak M. P. Zwaan R. A. (2007). Body posture facilitates retrieval of autobiographical memories. Cognition 102, 139–149. 10.1016/j.cognition.2005.12.009 16472550

[B13] Domahs F. Moeller K. Huber S. Willmes K. Nuerk H.-C. (2010). Embodied numerosity: implicit hand-based representations influence symbolic number processing across cultures. Cognition 116, 251–266. 10.1016/j.cognition.2010.05.007 20513381

[B14] Fischer M. H. Zwaan R. A. (2008). Embodied language: a review of the role of the motor system in language comprehension. Q. J. Exp. Psychol. 61, 825–850. 10.1080/17470210701623605 18470815

[B15] Gabbard C. Rabb C. Gentry V. (1998). Attentional stimuli and programming hand selection: a developmental perspective. Int. J. Neurosci. 96, 205–215. 10.3109/00207459808986468 10069620

[B16] Geschwind N. Galaburda A. (1984). Cerebral Dominance: The Biological Foundations. Cambridge: Harvard University Press.

[B17] Ghazanfar A. A. Turesson H. K. (2008). How robots will teach us how the brain works. Nat. Neurosci. 11:3. 10.1038/nn0108-338975469

[B18] Gold J. I. Shadlen M. N. (2000). Representation of a perceptual decision in developing oculomotor commands. Nature 404, 390–394. 10.1038/35006062 10746726

[B19] Gold J. I. Shadlen M. N. (2007). The neural basis of decision making. Annu. Rev. Neurosci. 30, 535–574. 10.1146/annurev.neuro.29.051605.113038 17600525

[B20] Gottlieb J. (2007). From thought to action: the parietal cortex as a bridge between perception, action, and cognition. Neuron 53, 9–16. 10.1016/j.neuron.2006.12.009 17196526

[B21] Gut M. Urbanik A. Forsberg L. Binder M. Rymarczyk K. Sobiecka B.. (2007). Brain correlates of right-handedness. Acta Neurobiol. Exp. 67, 43–51. 10.55782/ane-2007-1631 17474320

[B22] Hammond G. (2002). Correlates of human handedness in primary motor cortex: a review and hypothesis. Neurosci. Biobehav. Rev. 26, 285–292. 10.1016/S0149-7634(02)00003-9 12034131

[B23] Hicks R. E. Kinsbourne M. (1976). On the genesis of human handedness: a review. J. Mot. Behav. 8, 257–266. 10.1080/00222895.1976.10735080 23961928

[B24] Kim S. Ashe J. Hendrich K. Ellermann J. Merkle H. Ugurbil K. Georgopoulos A.. (1993). Functional magnetic resonance imaging of motor cortex: hemispheric asymmetry and handedness. Science 261, 615–615. 10.1126/science.8342027 8342027

[B25] Kimura D. (1961a). Cerebral dominance and the perception of verbal stimuli. Canad. J. Psychol. 15:166. 10.1037/h0083219

[B26] Kimura D. (1961b). Some effects of temporal-lobe damage on auditory perception. Canad. J. Psychol. 15:156. 10.1037/h0083218 13756014

[B27] Kimura D. (1983). Speech representation in an unbiased sample of left-handers. Hum. Neurobiol. 2:147. 6668232

[B28] Kimura D. (2011). From ear to brain. Brain Cogn. 76, 214–217. 10.1016/j.bandc.2010.11.009 21236541

[B29] King F. Kimura D. (1972). Left-ear superiority in dichotic perception of vocal nonverbal sounds. Canad. J. Psychol. 26:111. 10.1037/h0082420 5035120

[B30] Knox C. Kimura D. (1970). Cerebral processing of nonverbal sounds in boys and girls. Neuropsychologia 8, 227–237. 10.1016/0028-3932(70)90010-2 5535486

[B31] Kubanek J. Snyder L. H. Abrams R. A. (2015). Reward and punishment act as distinct factors in guiding behavior. Cognition 139, 154–167. 10.1016/j.cognition.2015.03.005 25824862 PMC4397189

[B32] Kubanek J. Snyder L. H. Brunton B. W. Brody C. D. Schalk G. (2013). A low-frequency oscillatory neural signal in humans encodes a developing decision variable. Neuroimage 83, 795–808. 10.1016/j.neuroimage.2013.06.085 23872495 PMC3815962

[B33] Markman A. Dietrich E. (2000). Extending the classical view of representation. Trends Cogn. Sci. 4, 470–475. 10.1016/S1364-6613(00)01559-X 11115761

[B34] Niedenthal P. M. (2007). Embodying emotion. Science 316, 1002–1005. 10.1126/science.1136930 17510358

[B35] Pfeifer R. Bongard J. (2006). How the Body Shapes the Way we Think: A New View of Intelligence. New York: MIT press. 10.7551/mitpress/3585.001.000135692490

[B36] Schall J. D. (2002). The neural selection and control of saccades by the frontal eye field. Philos. Trans. R. Soc. Lond. B, Biol. Sci. 357, 1073–1082. 10.1098/rstb.2002.1098 12217175 PMC1693021

[B37] Schluter N. Rushworth M. Passingham R. Mills K. (1998). Temporary interference in human lateral premotor cortex suggests dominance for the selection of movements. A study using transcranial magnetic stimulation. Brain 121, 785–799. 10.1093/brain/121.5.785 9619185

[B38] Schmitz J. Packheiser J. Birnkraut T. Hinz N.-A. Friedrich P. Güntürkün O. Ocklenburg S. (2019). The neurophysiological correlates of handedness: insights from the lateralized readiness potential. Behav. Brain Res. 364, 114–122. 10.1016/j.bbr.2019.02.021 30768993

[B39] Selen L. Shadlen M. Wolpert D. (2012). Deliberation in the motor system: reflex gains track evolving evidence leading to a decision. J. Neurosci. 32, 2276–2286. 10.1523/JNEUROSCI.5273-11.2012 22396403 PMC3299561

[B40] Serrien D. Ivry R. Swinnen S. (2006). Dynamics of hemispheric specialization and integration in the context of motor control. Nat. Rev. Neurosci. 7, 160–166. 10.1038/nrn1849 16429125

[B41] Solodkin A. Hlustik P. Noll D. Small S. (2001). Lateralization of motor circuits and handedness during finger movements. Eur. J. Neurol. 8, 425–434. 10.1046/j.1468-1331.2001.00242.x 11554905

[B42] Stancák Jr A. Pfurtscheller G. (1996). The effects of handedness and type of movement on the contralateral preponderance of μ-rhythm desynchronisation. Electroencephalogr. Clin. Neurophysiol. 99, 174–182. 10.1016/0013-4694(96)95701-6 8761053

[B43] Stins J. Kadar E. Costall A. (2001). A kinematic analysis of hand selection in a reaching task. Laterality 6, 347–367. 10.1080/713754421 15513181

[B44] Thelen E. Schöner G. Scheier C. Smith L. B. (2001). The dynamics of embodiment: a field theory of infant perseverative reaching. Behav. Brain Sci. 24, 1–34. 10.1017/S0140525X01003910 11515285

[B45] Treviño M. (2014). Stimulus similarity determines the prevalence of behavioral laterality in a visual discrimination task for mice. Sci. Rep. 4:7569. 10.1038/srep07569 25524257 PMC5378985

[B46] Treviño M. Castiello S. Arias-Carrión O. De la Torre-Valdovinos B. Coss y León R. M. (2021). Isomorphic decisional biases across perceptual tasks. PLoS ONE 16:e0245890. 10.1371/journal.pone.0245890 33481948 PMC7822501

[B47] Treviño M. Medina-Coss y León R. Haro B. (2020). Adaptive choice biases in mice and humans. Front. Behav. Neurosci. 14:99. 10.3389/fnbeh.2020.00099 32760255 PMC7372118

[B48] Treviño M. y León R. M.-C. (2020). Distributed processing of side-choice biases. Brain Res. 1749:147138. 10.1016/j.brainres.2020.147138 33002485

[B49] Tversky A. Kahneman D. (1981). The framing of decisions and the psychology of choice. Science 211, 453–458. 10.1126/science.7455683 7455683

[B50] Williams S. (1986). Factor analysis of the Edinburgh handedness inventory. Cortex 22, 325–6. 10.1016/S0010-9452(86)80058-2 3731804

[B51] Wilson M. (2002). Six views of embodied cognition. Psychon. Bull. Rev. 9, 625–636. 10.3758/BF03196322 12613670

[B52] Xie T. Adamek M. Cho H. Adamo M. A. Ritaccio A. L. Willie J. T. Brunner P.. (2024). Graded decisions in the human brain. Nat. Commun. 15:4308. 10.1038/s41467-024-48342-w 38773117 PMC11109249

